# The successful treatment of *Enterocytozoon bieneusi* Microsporidiosis with nitazoxanide in a patient with B-ALL: A Case Report

**DOI:** 10.3389/fcimb.2022.1072463

**Published:** 2023-01-09

**Authors:** Lanlan Zhou, Zebing Guan, Chaolun Chen, Qiuhua Zhu, Shiqiu Qiu, Yanan Liu, Mingjie Li, Wenbin Zeng, Hong Wang, Yanmin Gao, Yuemei Yuan, Hanling Zhang, Guanqiao Ruan, Xueyi Pan

**Affiliations:** Department of Hematology, The First Affiliated Hospital of Guangdong Pharmaceutical University, Guangzhou, China

**Keywords:** *Enterocytozoon bieneusi* infection, leukemia, nitazoxanide, metagenomic next-generation sequencing, case report

## Abstract

**Introduction:**

*Enterocytozoon bieneusi* (*E. bieneusi*) Microsporidia can cause opportunistic infections in immunocompromised patients and is also an emerging disease in these individuals. Its clinical manifestations are chronic diarrhea and severe wasting syndrome, these can be extremely debilitating and carry a significant risk of death for immunocompromised patients. Often, microsporidia cannot be confirmed immediately by routine examination and culture. Effective and available treatment options are limited for infections caused by *E. bieneusi* in humans. Such cases are very rare in Chinese Mainland.

**Case presentation:**

A 47-year-old male had recurrent, profuse watery diarrhea and abdominal discomfort for more than 7 months, with a fever for 5 days. Two years earlier, he received treatment with a modified BFM-90 protocol for acute B cell lymphoblastic leukemia and is currently in the final stages of maintenance therapy with oral methotrexate and mercaptopurine. The leukemia was assessed as still in remission two months ago. PET/CT showed massive peritoneal fluid accumulation and a high uptake area in the diffused peritoneum (SUVmax 12.57), suggesting tumor invasion or microbial infections. However, broad-spectrum antibacterial therapies were ineffective. Metagenomic sequencing of plasma and peritoneal fluid showed no suggestion of the existence of a tumor but instead showed a high sequence number of DNA and RNA of the Microsporidia. His albendazole treatment failed and subsequent treatment with nitazoxanide successfully resolved the infection.

**Conclusion:**

This case shows that we should consider the possibility of atypical pathogen infection in patients with hematologic malignancy who repeatedly develop unexplained diarrhea with wasting. mNGS can help rule out malignant neoplasms and diagnose infections. Our results suggest that nitazoxanide effectively treats *E. bieneusi* microsporidia infections.

## Introduction

Microsporidiosis is a frequent enteric infection affecting patients with AIDS and immunocompromised individuals worldwide (Checkley et al., 2015). *E. bieneusi* is the most common cause of microsporidiosis in humans. The most common clinical manifestation is diarrhea and weight loss (Kotler and Orenstein, 1998). Infections in normally immunocompetent mammals are usually chronic and asymptomatic, whereas immunodeficient hosts often develop fatal infections (Texier et al., 2010). Digestive symptoms caused by microsporidium infection in HIV-infected patients or organ transplant recipients have been widely reported (Desportes et al., 1985; Dieterich et al., 1994; Bicart-Sée et al., 2000; Pomares et al., 2012). However, there are few reports on microsporidiosis in patients with hematopoietic diseases. Although such cases have been reported sporadically all over the world, but very rare in Chinese Mainland. Traditional culture and routine stool detection are often negative, multiple detection methods are either indiscriminating microsporidia, time-consuming, or cannot be carried out in many health care settings (Han et al., 2021). There is no clear consensus for treatment of microsporidiosis.

We report a case of a 47-year-old patient with B-cell acute lymphoblastic leukemia (B-ALL) who presented with an *E. bieneusi* gastrointestinal infection diagnosed by peritoneal effusion mNGS, which was successfully treated with nitazoxanide.

## Case report

### Case presentation

A 47-year-old male was hospitalized on February 4, 2022, presenting with recurrent diarrhea for more than 7 months and with a fever for 5 days (highest temperature of 38.3°C). The patient reported about five yellow watery stools per day, accompanied by nausea, abdominal distension, and poor appetite. The patient had a weight loss of more than 10 kilograms in 6 months. The source of this patient’s microsporidium infection was unclear. For the past year, his travel had been limited to Guangdong Province in China. He was diagnosed with B-ALL in July 2019. He had previously received more than 10 antileukemia combination chemotherapies following the modified Berlin-Frankfurt-Munster 90 (BFM-90) protocol (Rajendra et al., 2021) for acute lymphoblastic leukemia, beginning in March 2020 with long-term oral methotrexate (MTX, 20 mg/m^2^, once a week) and 6-mercaptopurine (6-MP, 50 mg/m^2^, once a day) as maintenance chemotherapy. Approximately 1 month and 6 months after the initial symptoms of diarrhea, the patient underwent colonoscopy and gastroscopy, respectively, which showed no obvious abnormal lesions. During the period of diarrhea, repeated culture and routine detection of stool, several bone marrows punctures and a positron emission tomography/computed tomography (PET-CT) examination were performed, and no evidence of tumor recurrence was found.

At admission, the patient was dehydrated and had abdominal distention and pain; taking MTX and 6-MP at the time. Laboratory analyses showed lymphocytopenia (0.103×10^9^/L), with macrocytic and moderate anemia (hemoglobin concentration was 86 g/L, the mean erythrocyte volume was 113 fl), C-reactive protein level was 148 mg/L, procalcitonin was 0.272 ng/ml. His CD4 and CD19 counts were 31 cells/µl (24.3%) and 5 cells/µl (4%) in the peripheral blood, respectively. Stool routine, urine routine, stool culture, urine culture, blood culture, Widal test, waffian reaction, anti-nuclear antibody, anticardiolipin antibody, antineutrophil cytoplasmic antibody, G and GM test, T-spot TB test, EB virus, and cytomegalovirus DNA were all negative. The smear of the stool followed by Wright’s stain had also been performed, and a small number of spores were observed under a light microscope, but it could not confirm the species of spores. The liver and kidney functions were normal and the albumin level was 19.1 g/L. Bone marrow smear and flow cytometry examination showed no abnormalities.

Physical examination revealed that the patient had an unwell and anemic appearance, unpalpable superficial lymph nodes, abdominal distention, abdominal muscle tension, tenderness, shifting dullness, and slight edema of the lower limbs. The cardiopulmonary examination was unremarkable.

Based on these tests above, the diagnosis of local or systemic bacterial, viral, fungal and tuberculosis infection was not supported, nor was the evidence of tumor recurrence.

### Initial treatment

The patient immediately stopped taking oral chemotherapy for leukemia, was first administered piperacillin/tazobactam, amikacin and caspofungin, and was treated symptomatically with intravenous fluids, antidiarrheal, probiotics, folic acid and vitamin B12. Fever still occurred 5 days after treatment, the highest temperature was 38.4°C, and his diarrhea remained uncontrolled (approximately 5 liquid bowel movements per day). Abdominal distension, abdominal pain, and other related symptoms did not improve. Routine blood and biochemical examinations showed no apparent changes.

### PET-CT

**Figure 1:**
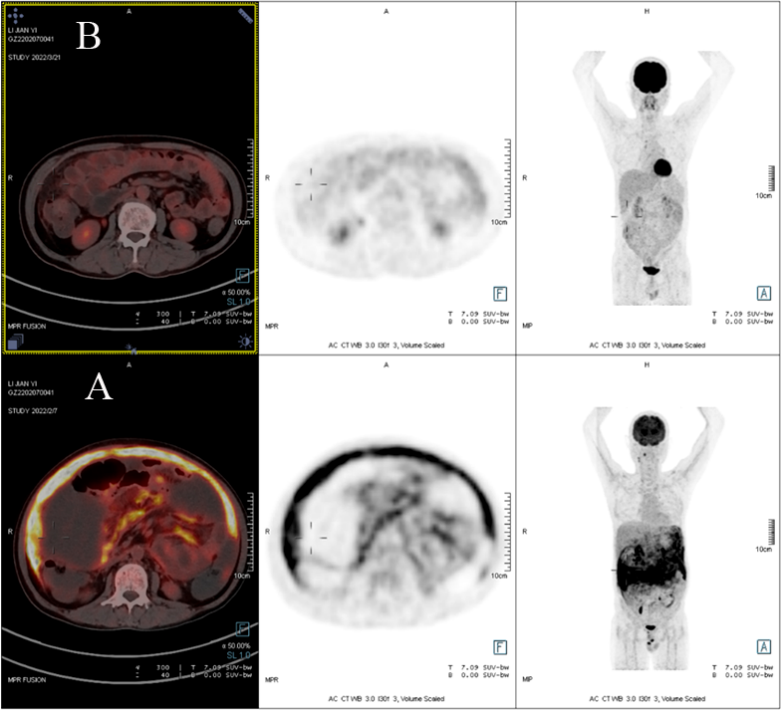
**(A)** PET scan demonstrating diffuse peritoneal areas of high glucose uptake before treatment. **(B)** PET/CT scan revealing the decline in glucose metabolism throughout the peritoneal region after treatment.

Due to a lack of response from the previous antibiotic treatment, the patient underwent another full-body PET-CT, which revealed elevated glucose metabolism throughout the peritoneum (standardized uptake value (SUV) max=12.57) and part of the penis (SUVmax=13.41) and massive peritoneal effusion with peritonitis, suggesting tumor or microbial infiltration (**Figure 1A**). Then,

### NGS testing

**Figure 2:**
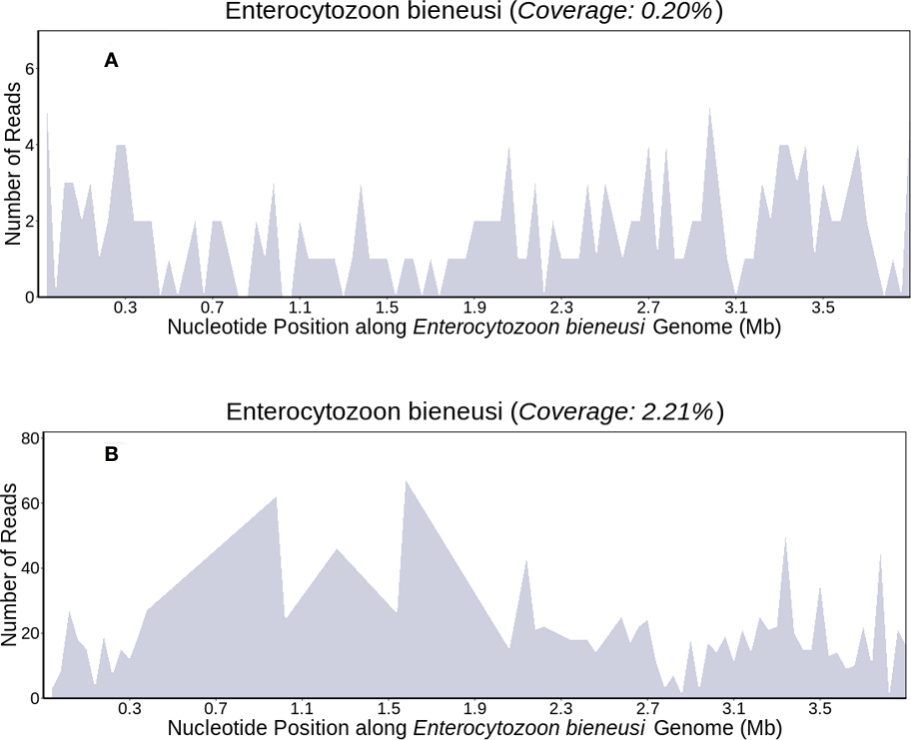
The patient’s ascites mNGS coverages mapped to (E. bieneusi) (DNA/RNA) **(A, B)**.

We performed peritoneal puncture drainage on the patient, peritoneal fluid was obtained from the patient for mNGS testing. *E. bieneusi* genomic sequences were detected, suggesting a likelihood of infectious disease. The sequencing results were obtained within 24 hours, which revealed E. bieneusi DNA sequence reads was 85, the RNA sequence reads was 3495,the DNA sequences covered 0.20% of the genome (**Figure 2A**), the RNA sequences covered 2.21% of the genome (**Figure 2B**). Meanwhile, target gene sequencing and mutational analysis showed no tumor-specific or germline mutations.

The patients were evaluated using 475 Lymphoma-related NGS panel. DNA extraction, sequencing library preparation, and targeted capture enrichment were carried out following the methods as previously described with modifications (Tong et al., 2019). Target enriched libraries were sequenced on the HiSeq4000 platform (Illumina). Single-nucleotide variants, indels, structural variants, and copy number changes were identified by validated bioinformatics process from paraffin-embedded tissues (DePristo et al., 2011; Amarasinghe et al., 2013; Newman et al., 2014; Shen and Seshan, 2016). Mutant allele frequency (MAF) cutoff for single-nucleotide variants and indels was defined as 1%. The log2 ratio cut-off for copy number gain was defined as 2.0 for tissue samples. A log2 ratio cut-off of 0.6 was used for copy number loss detection.

The DNA of ascitic fluid extracted using the TIANamp Magnetic DNA Kit (Tiangen) according to the manufacturer’s protocols. DNA libraries were prepared using the KAPA Hyper Prep kit (KAPA Biosystems) according to the manufacturer’s protocols and were 75bp single-end sequenced on Illumina NextSeq 550Dx (Illumina). We use in-house developed bioinformatics pipeline for pathogen identification. Our microorganism genome database contained bacteria, fungi, virus and parasite genomic sequences (download from https://www.ncbi.nlm.nih.gov/).

We used the following criteria for positive results of mNGS:

For Mycobacterium, Nocardia and Legionella pneumophila, the result was considered positive if a species detected by mNGS had a species-specific read number≥1. For bacteria (excluding Mycobacterium, Nocardia and Legionella pneumophila), fungi, virus and parasites, the result was considered positive if a species detected by mNGS had at least 3 non-overlapping reads. Pathogens detected in the negative ‘no-template’ control (NTC) were excluded but only if the detected reads was ≥10-fold than that in the NTC.

### Follow-up and outcomes

We attempted to treat the patient with albendazole at 400 mg twice daily for 7 days (14 February to 20 February 2022). The patient became weaker and continued to have severe diarrhea with worsening abdominal distension and pain. Bedside abdominal ultrasound indicated that there was still a large amount of ascites, and more separation bands were visible, which resulted in no peritoneal fluid draining from the abdominal drainage tube. Albendazole tablets probably failed to produce a clinical reaction.

After a review of extensive literature, alternatively, the patient took nitazoxanide (500 mg twice a day for 14 consecutive days) with reported successful treatment in case reports with AIDS and solid tumors after organ transplantation. About three days after the above treatment, his stool began to form and the frequency decreased; he presented no nitazoxanide-related side effects and was discharged home. The patient completed fourteen days of nitazoxanide. A full-body PET-CT was performed more than one month later, which showed a decline in glucose metabolism throughout the peritoneal and penile regions; meanwhile, the ascites had completely resolved (**Figure 1B**). His CD4 count increased to 297 cells/µl (26.3%) in peripheral blood, and his hemoglobin level was normal. He was successfully treated with nitazoxanide, and the clinical symptoms disappeared.

At the recent follow-up, the patient had no anemia, no fever, no diarrhea, no abdominal pain, no abdominal distension, a normal diet, and sleep. A whole-body contrast-enhanced CT was also obtained on July 11, 2022(approximately 5 months after treatment for Microsporidia), which also showed no abnormalities. Considering that the maintenance treatment of the patient’s leukemia was almost over, the patient was instructed to review the signs and examinations related to leukemia regularly.

## Discussion

There are approximately 14 species of microsporidia can infect humans, which are classified as a basic branch of fungi with Cryptococcus (Didier and Weiss, 2006). Microsporidia can affect multiple organ systems, including the bowel, eye, muscle, lung and kidney. *E. bieneusi* can cause diarrhea, abdominal pain, fever, nausea, malabsorption and weight loss (James et al., 2013; Han and Weiss, 2018). The infection has been described in both immunocompetent hosts and immunocompromised individuals (Al, 2017; Saffo and Mirza, 2019; Daniela et al., 2021; Kwon et al., 2021; Maillard et al., 2021).

According to previous reports, the main risk factor for microsporidiosis, especially in AIDS patients, is cell-mediated immune suppression (Sarfati et al., 2001), individuals with a CD4 T-lymphocyte count below 50 to 100 cells/mm3 are more susceptible to microsporidiosis (Halánová et al., 2019). Our patient had a low CD4 count and suppressed immune function due to long-term oral chemotherapy drugs, consistent with the above study results. Isolation of microsporidia by tissue culture is not helpful for routine clinical diagnosis, and examination of body fluid by light microscopy cannot confirm the species of microsporidia. Chromotrope 2R stains and fluorescent stains are remarkably useful for the diagnosis of microsporidia, however, they are all time-consuming procedures (Franzen and Müller, 1999; Thellier and Breton, 2008). mNGS, as a hypothesis-free and culture-free molecular technique, could also be used to identify the exact species of microsporidia known to cause human infections and be more sensitive for diagnosis (Chiu and Miller, 2019; Han et al., 2021). Our patient was eventually diagnosed with *E. bieneusi* when his peritoneal fluid was sent for mNGS testing for microsporidia; This indicates that when patients with malignant hematopoietic system tumors have atypical infections with persistent symptoms, mNGS can provide multiple diagnostic clues and is vital in the differential diagnosis of patients with suspected infection, especially in the case of empirical antibiotic treatment failure.

Many reports have revealed that the improvement of immune function can lead to a clinical response in patients with gastrointestinal microsporidiosis, as well as the elimination of the organism and normalization of the intestinal architecture (Miao et al., 2000; Martins et al., 2001). P Maggi et al. revealed that the remission of diarrhea seems to be related to an increased CD4+ cell count, not to the human immunodeficiency viral load (Maggi et al., 2000). However, our patient’s symptoms, including diarrhea and abdominal distension, became progressively worse after he stopped taking oral chemotherapy, which may endanger the patient’s life, and this means that anti-microsporidium therapy must be initiated.

In general, no current effective commercial therapy for *E. bieneusi*. Albendazole works mainly by inhibiting tubulin and has the most consistent activity against microsporidia (Han and Weiss, 2018). However, albendazole is not very effective against *E. bieneusi*, and unable to clear the *E. bieneusi* microsporidia (Dieterich et al., 1994; Shane et al., 2017). Our case did not respond to the drug either. Fumagillin is the current recommendation for treating *E. Bieneusi* infections in AIDS patients (Maillard et al., 2021) and patients with various organ transplants or stem cell transportation (Champion et al., 2010; Desoubeaux et al., 2013; Al, 2017). However, oral fumagillin is not available in China, and its use is limited due to its adverse effects of bone marrow toxicity (Molina et al., 2002). Meanwhile, Other inhibitors that could be used to treat microsporidiosis, such as those targeting adenosine triphosphate isomerase (TIM), chitin synthase, are under *in vitro* research or development (Han and Weiss, 2018). Nitazoxanide has been reported effective against *E. bieneusi* infection in HIV-infected people (Doumbo et al., 1997; Bicart-Sée et al., 2000), also successfully applied in the treatment of *E. bieneusi* infection in solid tumor organ transplant recipients (Pomares et al., 2012) and immunocompetent patients (Saffo and Mirza, 2019). After full consultation with the patient, we decided to trial nitazoxanide 500mg orally twice one day for 14 days, and this infection was eventually treated successfully. three-month follow-up with the patient revealed no infection relapse.

This report demonstrates that we should consider the microsporidia infection even in leukemia patients without hematopoietic stem cell transplantation when symptoms fail to resolve. *MNGS can help optimize the sensitivity of diagnosis when tumors and infections cannot be clinically distinguishable, and the effectiveness of nitazoxanide redounded to the diagnosis of the definitive E. Bieneusi infection, before the pathogen was confirmed by another method, such as the PCR test*. This case study also suggests that nitazoxanide, which is not the first-line treatment but has already been successfully used in a B-ALL patient without any side effects, especially bone marrow toxicity.

## Data availability statement

The data presented in the study are deposited in the Dryad repository, the unique digital object identifier (DOI): was "doi: 10.5061/dryad.c2fqz61cn".

## Ethics statement

Written informed consent was obtained from the individual(s) for the publication of any potentially identifiable images or data included in this article.

## Author contributions

LZ, ZG, CC, and XP were the primary physicians who provided patient diagnosis and treatment. QZ, YL, SQ, ML, WZ, YG, HW, YY, HZ, and GR collected and analyzed clinical data. LZ wrote the paper. All authors contributed to the article and approved the submitted version.
